# The SHA.LIN renal stone scoring system for predicting stone-free status and postoperative outcomes after percutaneous nephrolithotomy

**DOI:** 10.3389/fsurg.2025.1563801

**Published:** 2025-06-18

**Authors:** Lei Jia, Jiuyi Wang, Tao Wang, Shaogang Wang, Qiang Li, Qinzhang Wang, Jihong Liu, Kai Zeng

**Affiliations:** ^1^ Department of Urology, First Affiliated Hospital, Medical College, Shihezi University, Shihezi, Xinjiang, China; ^2^ School of Medicine, Shihezi University, Shihezi, Xinjiang, China; ^3^ Department of Urology, Tongji Hospital, Tongji Medical College, Huazhong University of Science and Technology, Wuhan, China

**Keywords:** urology, percutaneous nephrolithotomy (PCNL), scoring system, stone clearance, complications

## Abstract

**Objective:**

To introduce a novel renal stone scoring system (SHA.LIN) for percutaneous nephrolithotomy (PCNL) and to compare the predictive power of the SHA.LIN scoring system vs. the Guy's stone score and S.T.O.N.E. scoring system for postoperative outcomes.

**Methods:**

Six reproducible parameters available from preoperative computed tomography (CT) data were measured: stone burden (S), hydronephrosis (H), anatomical distribution (A), length of tract (L), indicator of CT (I), and number of involved calyces (N). Data from patients who underwent PCNL between May 2019 and January 2023 were retrospectively reviewed. Correlations between scores from the 3 systems (i.e., SHA.LIN, STONE, and Guy's) and stone-free status (SFS), hemoglobin change, estimated blood loss (EBL), operative time (OT), and postoperative length of hospital stay (PLOS) were evaluated using standard statistical methods.

**Results:**

The overall stone-free rate was 69.7% (248/356), and complications occurred in 111 (30.9%) patients. Patients were divided into 2 groups (stone-free vs. non-stone-free), with median (IQR) scores as follows: Guy's, 2 (1–2) vs. 3 (2–3); S.T.O.N.E., 6 (6–8) vs. 8 (7–9.5); and SHA.LIN, 7 (7–9) vs. 11 (10–12.5), respectively (*p* < 0.001 for all). Univariate regression analysis revealed that the three scoring systems were significantly associated with SFS and OT, although none were significantly correlated with PLOS. EBL and hemoglobin change were significantly correlated with the SHA.LIN score. Multivariate regression analysis revealed that the three scoring systems were significantly associated with SFS. EBL and hemoglobin change were significantly correlated with the SHA.LIN score. Receiver operating characteristic (ROC) curve analysis revealed that the three scoring systems demonstrated comparable predictive accuracy for SFS and complications, with SHA.LIN having the highest area under the ROC curve (AUC) (0.852 and 0.774, respectively). Analysis of the respective AUCs revealed that the SHA.LIN score more accurately predicted EBL (AUC 0.807) than the other 2 scoring systems.

**Conclusion:**

The SHA.LIN scoring system accurately predicted postoperative outcomes of PCNL and demonstrated potential as an adjuvant tool for surgical planning. The three scoring systems demonstrated strong associations with SFS, in addition, the SHA.LIN score was also significantly associated with the risk of surgical bleeding.

## Introduction

The prevalence of urolithiasis is approximately 1%–15% and varies with age, sex, diet, and geographical environment; epidemiological data indicate that its prevalence is increasing on a global scale (1). With the development of minimally invasive surgical techniques, surgical treatment for upper urinary tract stones has evolved from traditional open surgery to minimally invasive approaches. Among the treatment options for urolithiasis, the most recent recommendations from the European Association of Urology (EAU) guidelines recommend PCNLstone as the first-line surgical option for upper urinary tract stones with a diameter >20 mm (2). Although the PCNL procedure yields a higher stone-free status (SFS) than ureteroscopic lithotripsy (URS) and extracorporeal shock wave lithotripsy (ESWL), it also has a higher procedural complication rate, including infection, organ injury, bleeding requiring blood transfusion, and even risk of mortality (3, 4). The most important questions for urologists to consider before the procedure include surgical strategy and stratification of patient surgical risk factors, particularly in the context of improving surgical safety and increasing the stone-free rate. To address these problems, several renal stone scoring systems have been developed to provide information about stone complexity, reduce adverse outcomes, provide patients with proper operative counseling, and optimize surgery planning and decision-making (5–7). Guy's stone score, which is convenient to use, can provide fast and easy classification information of stones in four grades based on preoperative imaging and correlates well with complications and SFS. However, it does not take into account the density and size of renal stones. Okhunov et al. (5) developed the S.T.O.N.E. scoring system, consisting of five parameters: renal stone size, tract length, obstruction, number of involved calyces, and stone density. A higher score is correlated with a lower stone-free rate and greater surgical difficulty. However, the S.T.O.N.E. scoring system does not account for the distributional complexity of stone locations in the pelvicalyceal system and only includes the number of involved calyces and staghorn stone. Currently, however, there remains a lack of a universally accepted gold-standard scoring system among most investigators for the evaluation of stone complexity (8–10).

In a previous study, Chinese researchers conducted a systematic review of literature from 1976 to 2014 and integrated their clinical surgical experience to identify clinically significant and reproducible variables influencing the outcomes of PCNL. Based on these findings, the research team developed the SHA.LIN scoring system in 2015 and validated its effectiveness in predicting the SFS through subsequent research (11). However, there is a paucity of data supporting which system is best among the present scoring systems. External validation and comparison of the three scoring systems may ultimately facilitate the development of a more universal and widely accepted scoring system. As such, the aim of the present study was to introduce a new stone scoring system (SHA.LIN) and to compare its accuracy with the S.T.O.N.E. scoring system and Guy's stone score in predicting PCNL outcomes, including SFS and complications.

## Methods

### Ethics statement and SHA.LIN stone score

This study was approved by the Ethics Committee of Tongji Hospital, Tongji Medical College, Huazhong University of Science and Technology (Wuhan City, Hubei Province, China). The SHA.LIN stone score consists of six variables derived from preoperative non-contrast computed tomography (NCCT) and three-dimensional (3D) reconstruction. In traditional Chinese medicine, SHA.LIN refers to urolithiasis. The six reproducible variables include stone burden (S), hydronephrosis (H), anatomical distribution (A), length of tract (L), indicator of CT (I), and number of involved calyces (N). The stone burden is calculated by combining the measurements of the longest width and length of stones in square millimeters (mm^2^). It is scored from 1 to 4 points based on cross-sectional areas: 0–399 mm^2^, 400–799 mm^2^, 800–1,599 mm^2^, and ≥1,600 mm^2^, respectively. Hydronephrosis is scored based on the degree of dilatation of the renal pelvis (12, 13). No caliceal or pelvic dilatation is assigned 1 point; pelvic dilatation or caliceal dilatation is assigned 2 points; and severe caliceal dilatation or accompanied by renal parenchymal atrophy is assigned 3 points. The third variable is the distributional complexity of stone locations within the pelvicalyceal system. Stones in the renal pelvis or mid/lower calyx are assigned 1 point; stones in the upper calyx are assigned 2 points; stones in the calyceal diverticulum or partial staghorn stone are assigned 3 points; and a full staghorn stone is assigned 4 points. The length of the tract is measured as the distance from the skin to the center of the stone on the NCCT film. The fifth variable is the CT value of the stone (in HU), which represents the stone density. The last variable is the number of renal calyces containing stones. The specific assignment of each variable is summarized in Table 1. The total stone burden is the sum of all stone cross-sectional areas. Stone density is evaluated using a circular region of interest incorporated into the largest stone area on NCCT images. The score ranges from a minimum of 6 to a maximum of 17, with a higher score denoting a more complex stone.

**Table 1:** Summary of SHA.LIN stone score.

variable	Score			
	1	2	3	4
Stone size (mm^2^)	0–399	400–799	800–1,599	≥1,600
Hydronephrosis	None or mild	Moderate	Severe	
Anatomic	Pelvis or mid/lower calyx	Upper calyx	Calyceal diverticulum or partial staghorn stone	Staghorn stone
Length of tract (mm)	≤100	>100		
Indicator of CT (Hu)	≤950	>950		
Number of involved calices	0–2	≥ 3		

CT, computed tomography; HU, Hounsfield units.

### Clinical information

Data from patients who underwent PCNL for renal stones between May 2019 and January 2023 were retrospectively collected and analyzed. Exclusion criteria included age <18 years and no available preoperative NCCT data. Patients were excluded if they had undergone percutaneous nephrostomy tube or ureteral double-J stent placement before surgery. Patients who had any open, endoscopic, or laparoscopic surgery on the ipsilateral kidney were also excluded. One side was chosen at random for patients who underwent bilateral PCNL. For patients undergoing staged surgery, only the first-stage procedure was selected. All patients underwent laboratory investigations, including complete blood count, coagulation parameters, serum creatinine, plasma electrolytes, urinalysis, and urine culture with sensitivity testing, as well as imaging examinations, such as kidney-ureter-bladder (KUB) x-ray, urinary tract ultrasound, intravenous pyelogram, and CT. Demographic characteristics, including age, sex, body mass index (BMI), stone burden, location, operative time (OT), postoperative length of hospital stay (PLOS), estimated blood loss (EBL), stone-free status (SFS), and postoperative complications, were collected and analyzed. EBL was measured by subtracting the volume of irrigation fluids used from the total volume of collected fluids (blood + irrigation fluid) (14). Patients were categorized into two groups based on a blood loss cut-off value of 250 ml (14). Complications were graded in accordance with the modified Clavien-Dindo classification system (3).

**Figure 1:**
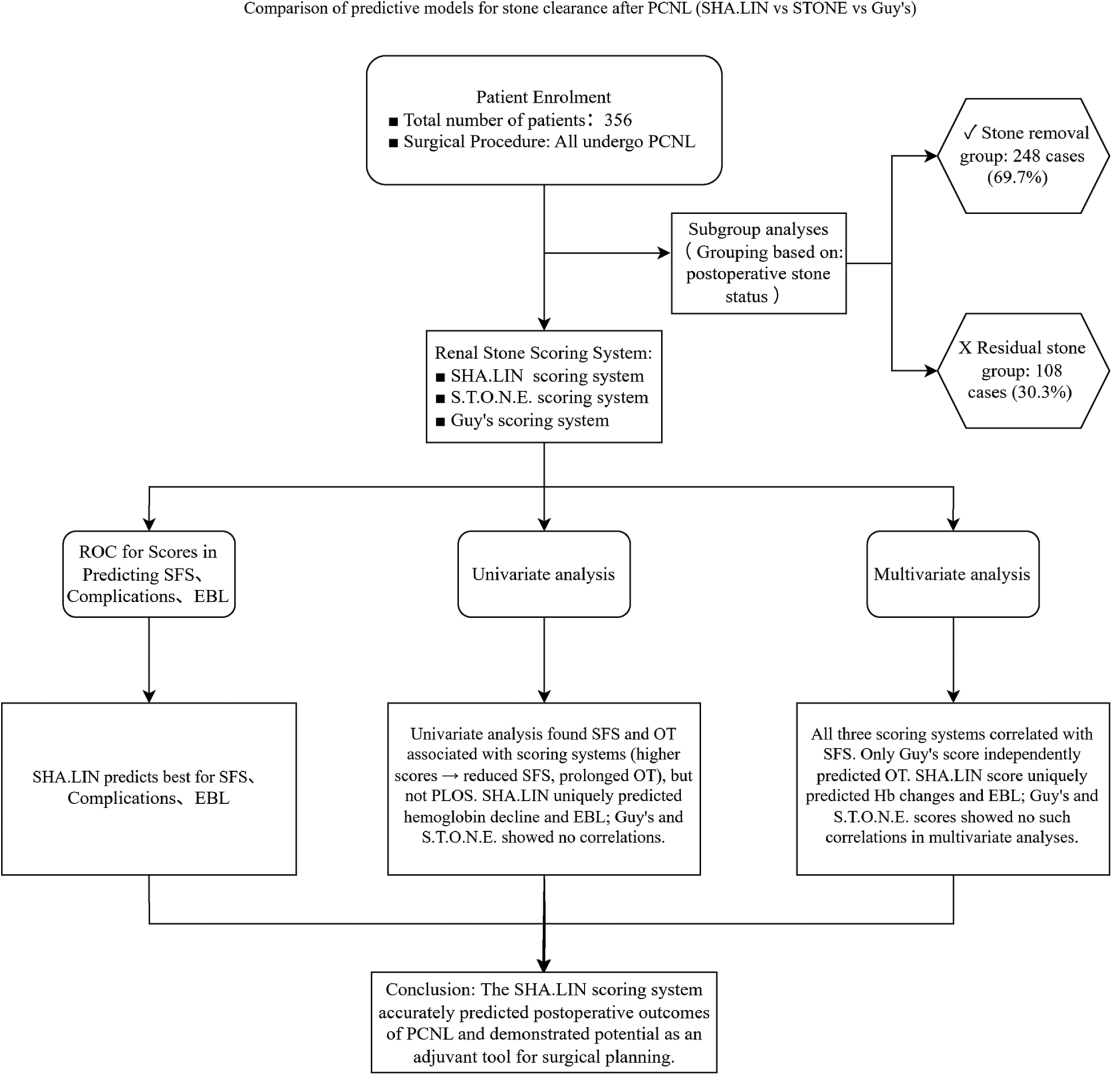
Research flowchart. This flowchart delineates the step-by-step systematic evaluation process of three predictive models, namely the SHA-LIN, S.T.O.N.E., and Guy’s scoring systems, concerning their ability to predict post-percutaneous nephrolithotomy (PCNL) outcomes. These outcomes include stone-free status (SFS), complications, estimated blood loss (EBL), and operative time (OT). The study employs both univariate and multivariate analyses to identify independent predictors associated with these outcomes.

All patients underwent preoperative scanning with a Siemens SOMATOM Force Dual-Source CT, with the scanning range covering the middle and lower abdomen. The scanning parameters were as follows: tube voltage 120 kV, tube current 125 mAs, and slice thickness 3 mm (15). The CT images were first adjusted to the abdominal window, and the slice showing the largest cross-sectional area of the stone was selected. The CT values were measured at one point in the central region of the stone and four surrounding points, and the average CT value was calculated as (the sum of the CT values at the five points)/5. Image reconstruction was performed using the Siemens syngo.via software tool. The scores from the three scoring systems were calculated as previously described by two experienced urologists: Guy's score (grades I, II, III, and IV) and SHA.LIN scores (6–8, 9–11, 12–14, and 15–17) were divided into four groups each, and S.T.O.N.E. scores into three groups (5–6, 7–8, and 9–13). All patients underwent KUB and ultrasound examinations every month for three months to detect SFS. The specific procedure of this study is illustrated in Figure 1.

### Surgical technique

Patients underwent PCNL under general anesthesia while positioned prone. After a target calyx was identified via ultrasound, the nephrostomy needle was accurately positioned under real-time ultrasound guidance, and a guide wire was inserted. Tract dilation was then performed through a percutaneous catheter, which gradually dilated the access tract and established a working tract. The size of the working tract was generally 24-Fr. An 18-Fr standard nephroscope was used in all patients. Fragmentation was performed using pneumatic methods or lithotripsy. A DJ stent was routinely implanted, and a 14-Fr nephrostomy tube was placed in the renal collecting system at the end of the procedure. KUB was utilized to evaluate postoperative SFS, and a residual stone with a diameter <4 mm was defined as SFS (16, 17).

### Statistical analysis

Categorical variables were expressed as numbers and percentages and were compared using the chi-squared test (or Fisher's exact test, where appropriate). Continuous variables were expressed as means and standard deviation (SD) and were compared using the independent samples *t*-test. The Mann–Whitney *U*-test was used to compare non-normally distributed continuous variables. Univariate and multivariate logistic regression analysis were used to assess the possible association between three stone scoring systems and SFS and EBL. Univariate and multivariate linear regression analysis were used to examine the possible association between three stone scoring systems and hemoglobin change, OT and PLOS. The following covariates were adjusted for in the multivariate regression analysis: (1) Demographic factors (Age, Gender, BMI); (2) Stone characteristics (Stone size, Stone HU, Number of stone, Side); (3) Operative parameters (Anatomic, Length of tract, Hydronephrosis). Receiver operating characteristic (ROC) curves were drawn to assess the predictive value of the three scoring systems on postoperative outcomes. The area under the ROC curve (AUC) was calculated for each stone score. Differences with *p* < 0.05 were considered statistically significant. Statistical analyses were performed using SPSS version 21.0 (IBM Corp., Armonk, NY, USA) for Windows (Microsoft Corp., Redmond, WA, USA) and R version 4.3.3 (2024-02-29).

## Results

Data from 356 patients were used to assess the three stone scoring systems investigated in this study. All patients underwent successful PCNL. Demographic and clinical data of the patients were summarized in Table 2. All patients were divided into two groups based on postoperative SFS (stone-free vs. non-stone-free); the stone-free rate was 69.7% (248/356). No significant differences were found between the two patient groups in terms of age, sex, BMI, stone laterality, change(s) in hemoglobin level, OT, and PLOS. Among the six potential variables in the SHA.LIN scoring system, an increased tract length (*p* = 0.003), increased stone size (*p* < 0.001), and stone anatomical location in the renal pelvis (*p* = 0.005) were associated with residual stones. However, differences in the number of stones (*p* = 0.064), stone density (*p* = 0.311), and degree of hydronephrosis (*p* = 0.642) were not statistically significant for residual stones. The three stone scoring systems were significantly associated with SFS. In the stone-free vs. non-stone-free group, the respective median (IQR) scores were as follows: SHA.LIN, 7 (7–9.5) vs. 11 (10–12.5); Guy's, 2 (1–2) vs. 3 (2–3); and S.T.O.N.E., 6 (6–8) vs. 8 (7–9.5) (*p* < 0.001 for all).

**Table 2:** Patient demographic and clinical data.

Variable	Stone-free (*n* = 248)	Non stone-free (*n* = 108)	*p* Value
Age, mean (SD)	49.3 ± 11.535	48.44 ± 11.103	0.449#
Gender (%)			
Male	144 (58.1)	71 (65.7)	0.336*
Female	104 (41.9)	37 (34.3)	
BMI (kg/m^2^)	24.4 (22.0–26.8)	24.3 (22.0–26.75)	0.901*
Side(%)			
Left	121 (48.8)	61 (56.5)	0.111
Right	127 (51.2)	47 (43.5)	
Stone burden (mm^2^), median(IQR)	1,015 (774–1,104)	1,111 (876–1,616)	<0.001##
Stone HU, median(IQR)	897 (680–1,107)	986 (742–1,099)	0.311##
Hydronephrosis (%)			
None or mild	173 (69.8)	70 (64.8)	0.642*
Moderate	54 (21.8)	28 (25.9)	
Severe	21 (8.5)	10 (9.3)	
Anatomic (%)			
Pelvis or mid/lower calyx	145 (58.4)	44 (40.7)	0.005*
Upper calyx	52 (21.0)	28 (26.0)	
Calyceal diverticulum or partial staghorn stone	35 (14.1)	19 (17.6)	
Staghorn stone	16 (6.5)	17 (15.7)	
Number of stone			
0–2	142 (57.3)	50 (46.3)	0.064*
≥3	106 (42.7)	58 (53.7)	
Length of tract (%)			
< 10 cm	153 (61.7%)	49 (45.4)	0.003*
>10 cm	95 (38.3)	59 (54.6)	
Hemoglobin change (mg/dl), median(IQR)	1.9 (1.3–2.3)	2.1 (1.5–2.3)	0.082##
Estimated Blood loss (%)			
> 250 cc	38 (15.3)	35 (32.4)	0.001*
< 250 cc	210 (84.7)	73 (67.6)	
OT (min)	70 (60–80)	78 (60–93)	0.163##
PLOS (d)	7 (6–9)	7.5 (6–9)	0.928##
Complications	57 (22.9%)	54 (50.0%)	<0.001##
Guy's score	2 (1–2)	3 (2–3)	<0.001##
S.T.O.N.E. score	6 (6–8)	8 (7–9.5)	<0.001##
SHA.LIN score	7 (7–9)	11 (10–12.5)	<0.001##

# *t*-test.

## Mann–Whitney-*U*-test.

* Chi squared test; SD, standard deviation; IQR, interquartile range; BMI, body mass index; HU, Hounsfield units; EBL, estimated blood loss; OT, operation time; PLOS, postoperative length of hospital stay;.

**Figure 2:**
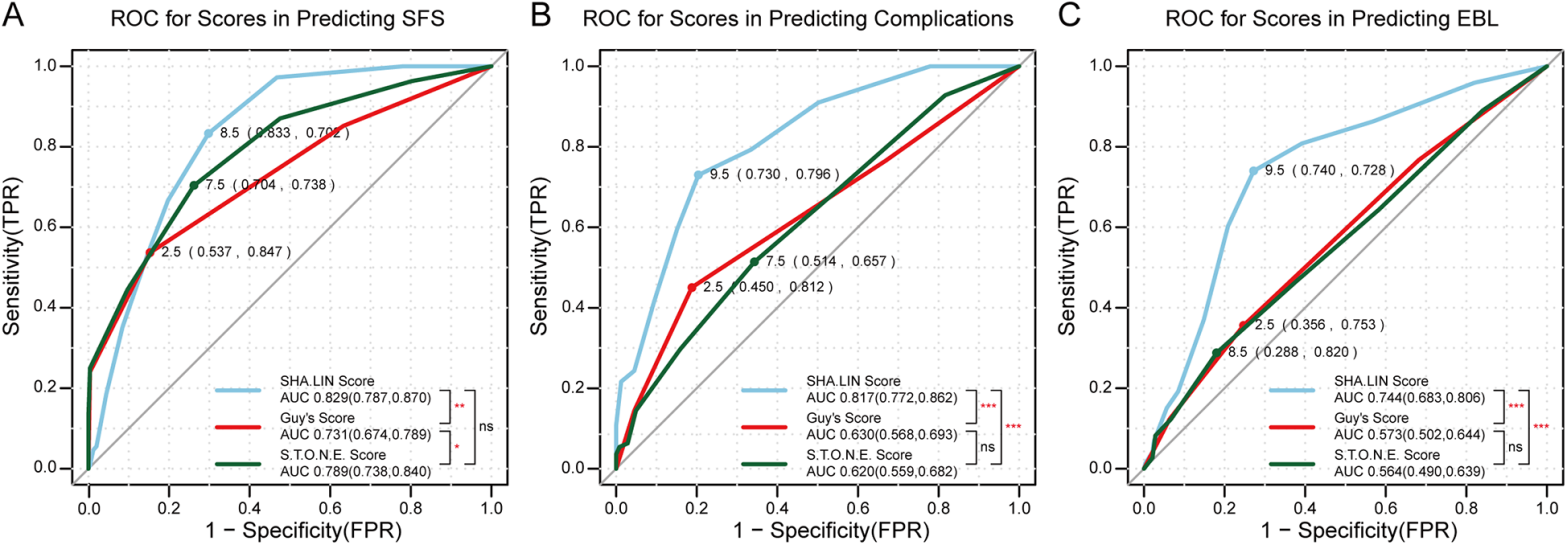
**(A)** ROC for scores in predicting SFS (stone-free status); **(B)** ROC for scores in predicting complications; **(C)** ROC for scores in predicting EBL (estimated blood loss). *p* values denoted as follows: ns (non-significant, *p* ≥ 0.05), *p < 0.05, **p < 0.01, and ***p < 0.001. The points on the curves represent the optimal cut-off values for each score, with their corresponding sensitivity and specificity rates. The area under the curve (AUC) values and their 95% confidence intervals (CI) are provided in the bottom right corner.

There was a positive association among the three stone scoring systems and complications and SFS (Table 3). The AUCs for the three stone scoring systems in predicting SFS are presented in Figure 2A. The SHA.LIN score yielded the highest accuracy in predicting SFS. The estimated AUC for the SHA.LIN stone score was 0.829 (95% CI: 0.787, 0.870), compared with 0.789 (95% CI: 0.738, 0.840) and 0.731 (95% CI: 0.674, 0.789) for the S.T.O.N.E. scoring system and Guy's stone score, respectively. According to the Clavien-Dindo classification, 111 patients experienced postoperative complications, distributed as follows: grade I (*n* = 49), grade II (*n* = 38), grade III (*n* = 21), and grade IV (*n* = 3). As the scores from the 3 stone scoring systems increased, so did the incidence of complications. Analysis of the respective AUCs (Figure 2B) demonstrated that the SHA.LIN stone score in predicting postoperative complications yielded an AUC of 0.817 (95% CI: 0.772–0.862), greater than those of the Guy's stone score [0.630 (95% CI: 0.568–0.693)] and S.T.O.N.E. [0.620 (95% CI: 0.559–0.682)]scoring systems.

**Table 3:** Stone free and complication rates of three stone score.

Stone scores	No. stone free/total (%)	No. complications/total (%)
Guy's score		
I	91/107 (85.0)	26/107 (24.3)
II	119/153 (77.8)	35/118 (29.7)
III	37/69 (53.6)	34/69 (49.3)
IV	1/27 (3.7)	16/27 (59.3)
S.T.O.N.E. score		
5–6	130/144 (90.3)	34/145 (23.4)
7–8	94/140 (67.1)	44/140 (31.4)
9–13	24/72 (33.3)	33/72 (45.8)
SHA.LIN score		
6–8	174/186 (93.54)	23/186 (12.3)
9–11	53/101 (52.47)	45/101 (41.6)
12–14	18/57 (31.5)	37/57 (64.9)
15–17	3/12 (25.0)	9/12 (75.0)

When the three stone scoring systems were individually assessed for their association with SFS, Hemoglobin change, EBL, OT, and PLOS (Tables 4, 5), univariate analysis revealed significant associations with SFS (Guy's: B-coefficient = −1.159, *p* < 0.001; S.T.O.N.E.: B-coefficient = −0.800, *p* < 0.001; SHA.LIN: B-coefficient = −0.560, *p* < 0.001) and OT (Guy's: B-coefficient =4.715, *p* < 0.001; S.T.O.N.E.: B-coefficient = 1.837, *p* = 0.005; SHA.LIN: B-coefficient = 0.854, *p* = 0.044). Higher scores correlated with reduced SFS rates and prolonged OT. None of the scoring systems showed a statistically significant association with PLOS (Guy's: B-coefficient = 0.160, *p* = 0.457; S.T.O.N.E.: B-coefficient = −0.028, *p* = 0.806; SHA.LIN: B-coefficient = 0.044, *p* = 0.546). The Guy's score was uniquely predictive of EBL (B-coefficient = −0.309, *p* = 0.032). The SHA.LIN score was uniquely predictive of both hemoglobin change (B-coefficient = 0.083, *p* < 0.001) and EBL (B-coefficient = −0.309, *p* < 0.001). In contrast, S.T.O.N.E. score exhibited no significant correlations with these parameters. Furthermore, multivariate analysis revealed significant associations with SFS for all three scoring systems (Guy's: B-coefficient = −0.660, *p* = 0.002; S.T.O.N.E.: B-coefficient = −0.559, *p* < 0.001; SHA.LIN: B-coefficient = −0.468, *p* < 0.001), with only the Guy's score uniquely predictive of OT (B-coefficient = 3.901, *p* = 0.008). The SHA.LIN score uniquely predictive of both hemoglobin change (B-coefficient = 0.094, *p* < 0.001) and EBL (B-coefficient = −0.313, *p* < 0.001); Guy's and S.T.O.N.E. scores showed no significant correlations with these parameters in multivariate analysis. Analysis of the respective AUCs revealed that the SHA.LIN stone score more accurately predicted blood loss [AUC 0.744 (95% CI: 0.683–0.806)] compared with the Guy's [AUC 0.573 (95% CI: 0.502–0.644)]and S.T.O.N.E. [AUC 0.564 (95% CI: 0.490, 0.639)] scores (Figure 2C).

**Table 4:** Univariate regression analysis of three stone scoring systems with stone-free status, hemoglobin change, EBL, OT, and PLOS.

Stone scores	B-coefficient	Odds-ratios	95% CI		*p* Value
			Lower	Upper	
Stone-free status					
Guy's score	−1.159	0.314	0.231	0.427	<0.001*
S.T.O.N.E. score	−0.800	0.450	0.370	0.547	<0.001*
SHA.LIN score	−0.560	0.571	0.505	0.647	<0.001*
EBL (ml)					
Guy's score	−0.309	0.734	0.554	0.973	0.032*
S.T.O.N.E. score	−0.144	0.866	0.747	1.003	0.056*
SHA.LIN score	−0.309	0.734	0.663	0.812	<0.001*
Hemoglobin change (mg/dl)					
Guy's score	0.077		−0.002	0.156	0.056#
S.T.O.N.E. score	0.024		−0.018	0.066	0.264#
SHA.LIN score	0.083		0.058	0.109	<0.001#
OT (min)					
Guy's score	4.715		2.330	7.100	<0.001#
S.T.O.N.E. score	1.837		0.556	3.119	0.005#
SHA.LIN score	0.854		0.026	1.682	0.044#
PLOS (d)					
Guy's score	0.160		−0.260	0.580	0.457#
S.T.O.N.E. score	−0.028		−0.252	0.196	0.806#
SHA.LIN score	0.044		−0.099	0.188	0.546#

* Logistic regression analysis.

# Linear regression analysis; CI, confidence interval; EBL, estimated blood loss; OT, operation time; PLOS, postoperative length of hospital stay.

**Table 5:** Multivariate regression analysis of three stone scoring systems with stone-free status, hemoglobin change, EBL, OT, and PLOS.

Stone scores	B-coefficient	Odds-ratios	95% CI		*p* Value
			Lower	Upper	
Stone-free status					
Guy's score	−0.660	0.517	0.344	0.777	0.002*
S.T.O.N.E. score	−0.559	0.572	0.438	0.746	<.001*
SHA.LIN score	−0.468	0.626	0.544	0.722	<.001*
EBL (cc)					
Guy's score	−0.025	0.975	0.678	1.403	0.893*
S.T.O.N.E. score	0.090	1.094	0.885	1.352	0.404*
SHA.LIN score	−0.313	0.731	0.653	0.820	<.001*
Hemoglobin change (mg/dl)					
Guy's score	0.042		−0.048	0.133	0.362#
S.T.O.N.E. score	−0.053		−0.106	0.000	0.052#
SHA.LIN score	0.094		0.064	0.123	<.001#
OT (min)					
Guy's score	3.901		1.026	6.775	0.008#
S.T.O.N.E. score	0.856		−0.834	2.546	0.321#
SHA.LIN score	−0.036		−0.969	0.898	0.940#
PLOS (d)					
Guy's score	0.138		−0.364	0.639	0.591#
S.T.O.N.E. score	−0.072		−0.367	0.224	0.635#
SHA.LIN score	0.055		−0.108	0.218	0.505#

* Logistic regression analysis.

# Linear regression analysis; CI, Confidence interval; EBL, estimated blood loss; OT, operation time; PLOS, postoperative length of hospital stay; The following covariates were adjusted for in the multivariate regression analysis: (1) Demographic factors (Age, Gender, BMI); (2) Stone characteristics (Stone size, Stone HU, Number of stone, Side); (3) Operative parameters (Anatomic, Length of tract, Hydronephrosis).

## Discussion

PCNL is well established as the first-line treatment for complex and high-volume upper urinary tract stones, with stone clearance rates ranging from 60% to 90% (18, 19). The ultimate goal of the procedure is to achieve SFS with minimal morbidity. However, as with all other surgical interventions, PCNL carries varying risks for complications and residual stones. Preoperative stratification of risk factors and reliable surgical planning remain of utmost priority for patients and urologists, particularly in the context of weighing the benefits of the procedure against potential risks and adverse effects. An increasing number of investigators have taken advantage of perioperative factors to predict SFS and the risk of complications after PCNL. To date, multiple attempts have been made to develop a scoring system that benefits patients. However, none of the proposed scoring systems have been widely adopted as the standard due to their limited predictive accuracy, lack of validation, and clinical utility.

In a previous study, Chinese investigators proposed the SHA.LIN stone score to assess the stone-free rate in PCNL and investigated its clinical utility in patients undergoing PCNL. However, unlike the Guy's stone score or S.T.O.N.E. scoring systems, the SHA.LIN scoring system has not been universally familiar to urologists and is still pending validation. The present study aimed to introduce the SHA.LIN score and compare its accuracy with that of the S.T.O.N.E. scoring system and Guy's stone score in predicting postoperative outcomes. Compared with previously proposed stone scoring systems, the SHA.LIN scoring system uses variables that are easily calculated from NCCT data and do not require specialized software. The stone score variables are defined based on operative experience and draw on extensive literature reviews and existing stone scoring systems (5–7).

The predictive accuracy of the Guy's stone score and S.T.O.N.E. scoring systems has been summarized and compared in published works; however, the results have varied. The Guy's stone score is reproducible and is simple to apply in routine clinical practice for assessing surgical risk. However, it does not account for critical variables such as stone burden, calyceal involvement, and stone density (6). Most research has reported that these parameters have an important influence on postoperative outcomes (10, 17, 20). In addition, the Guy's stone score has 4 grades, limiting the ability to evaluate the complexity of stone characteristics. Although the S.T.O.N.E. and SHA.LIN scores have common parameters, such as stone burden, tract length, degree of hydronephrosis, and stone essence, the definitions of these parameters remain different (5). For example, stone burden is an essential parameter in 2 of the scoring systems, whereas in the SHA.LIN scoring system, stone burden is estimated by combining stone length and maximum length in CT slice(s) in mm^2^. If there are multiple stones, the SHA.LIN score calculates the sum of the area of every stone. The S.T.O.N.E. score only calculates the largest stone by combining the measures of length and width in mm^2^. We believe that stone burden in the SHA.LIN scoring system can better reflect the complexity of stone characteristics. In the S.T.O.N.E. score, the definition of calyx and imaging plane is not standardized. Stone size and number of calyces involved are also not standardized and vary among different observers (8, 9). The hydronephrosis degree score is subjective in nature and does not have a clear definition in the S.T.O.N.E. score (8). In the SHA.LIN score, the authors not only refined the number of calyces containing stones, anatomical distribution of stones, and number of involved calyces but also provided a clear definition of each variable. Thus, urologists can perform a standardized evaluation of every patient using a CT scan, increasing the reliability of the outcome assessments.

In the present study, the stone clearance rate using PCNL was 69.3%, which is similar to rates reported by Krishnendu et al. (17) (71.5%), Thomas et al. (6) (62.0%), and Labadie et al. (21) (56.0%). Stone burden is the most crucial variable for predicting the SFS. In our study, there was a statistically significant difference in the mean size of stones in the 2 groups (*p* < 0.001). The presence of stones in multiple calyces was significantly associated with a decreased stone-free rate compared with single calyceal involvement. Staghorn stones have a significant association with SFS, with partial staghorn stones (56.7%) and complete staghorn stones (48.5%). Labadie et al. (21) reported that staghorn renal stones demonstrated a 40% stone-free rate among operated patients. In the Guy's stone score, with partial staghorn as grade III and complete staghorn as grade IV, stone clearance rates were 35% and 25%, respectively (6). Research has shown that stone distribution and location have an essential impact on SFS. There are 2 opposing opinions in the determination of stone distribution in the S.T.O.N.E. and Guy's scores (8). The Guy's stone score similarly assigns categorizations according to anatomical distribution in the renal pelvis, and lower, middle, and upper calyces. In contrast, the S.T.O.N.E. score prioritizes the number of stone-involved calyces, with an overall algorithm determining how much weight each location contributes to complications and SFS (8). In developing the SHA.LIN scoring system, the above 2 scoring systems were referenced. The authors not only considered the effect of staghorn stones on the stone clearance rate but also redefined the distribution of stones in the renal calyx. Stones in the renal pelvis or mid/lower calyx are assigned 1 point; stones in the upper calyx are assigned 2 points; stones in a calyceal diverticulum or partial staghorn stone are assigned 3 points; and full staghorn stones are assigned 4 points. We believe this classification method more accurately reflects the complexity of stone characteristics. In the present study, scores from the three scoring systems were significantly associated with SFS and OT. These conclusions are consistent with previous reports (10, 22, 23). We noted the comparable accuracy of the SHA.LIN score (AUC 0.829), Guy's score (AUC 0.731), and S.T.O.N.E. score (AUC 0.789) for predicting SFS. These results can be interpreted as SHA.LIN having a higher predictive power for SFS after PCNL than the other 2 scoring systems.

Bleeding is one of the most unpredictable and threatening complications during PCNL. Published data have shown that stone burden, degree of hydronephrosis, and staghorn stones are associated with an increased risk of blood loss complications (24). The variables of the SHA.LIN score include these risk factors. In recent studies, the relationship between EBL and stone scores was unclear (14, 17). In a study involving 117 patients, Akhavein et al. (25) reported that the S.T.O.N.E. score was significantly correlated with SFS; however, no such correlation was found with EBL. A previous study involving 437 patients (14) found no significant correlation between the S.T.O.N.E. score and EBL. In a study involving 246 patients who underwent PCNL, Labadie et al. (26) concluded that the Guy's and S.T.O.N.E. scores demonstrated a significant correlation with EBL. The differences in these studies may be explained by a low number of patients with renal stones or poor universality of the scoring systems. In the present study, EBL and hemoglobin change were significantly correlated with the SHA.LIN stone score. ROC curve analysis demonstrated a more accurate prediction of blood loss based on the SHA.LIN stone score compared with the other 2 stone scores. However, this conclusion needs further validation.

Although the SHA.LIN scoring system demonstrates high accuracy in predicting SFS and can be used to assess surgical risk factors, its clinical applicability remains limited by several shortcomings. First, the study predominantly relies on a single-center data bank and small sample size, without multicenter validation. Second, in China, the SHA.LIN scoring system is the first proposal of a predictive method for SFS after PCNL. We only compared it with the Guy's and S.T.O.N.E. scoring systems, not with the Clinical Research Office the Endourological Society nephrolithometric nomogram. Third, the scoring was independently performed by two urologists, during which random errors and inter-observer variations were present. Furthermore, we did not use CT to randomly detect SFS in all patients, as in other studies (5, 21). CT is a particularly accurate diagnostic method, with sensitivity and specificity ranging from 94% to 100% and 92% to 94.2% for kidney stones, respectively (27, 28). But, the sensitivity and specificity of KUB radiography is 44%–77% and 80%–87%, in detecting ureteric and renal stones respectively (29). As a result, even though we had all patients undergo KUB and ultrasound examinations monthly for 3 months, which may also have introduced bias in the SFS calculation. Finally, the scoring system does not integrate machine learning models, rendering it less competitive compared to emerging predictive tools. Additional work by Shabaniyan et al. demonstrated that decision support systems (DSS) based on classification methods can predict PCNL outcomes with up to 94.8% accuracy, while Hameed et al. achieved 81% accuracy in predicting stone-free status after PCNL for the challenging subset of staghorn calculi using Random Forest-based machine learning (30). These advances highlight the growing utility of machine learning in improving predictive accuracy for stone-free status beyond what conventional scoring systems can achieve. Addressing these limitations will advance traditional scoring systems toward intelligent and precise evolution, ultimately achieving personalized surgical risk stratification and optimized postoperative outcomes.

## Conclusion

The SHA.LIN scoring system may be used to predict postoperative SFS in PCNL and may demonstrate its potential as an adjuvant tool for surgical planning and patient counseling. Furthermore, the three stone scoring systems-namely, Guy's, S.T.O.N.E., and SHA.LIN-demonstrated an association with SFS; in addition, the SHA.LIN score was also significantly associated with the risk of surgical bleeding. Nevertheless, further validation of the SHA.LIN scoring system using external data is important to confirm its accuracy and general applicability for PCNL.

## Data Availability

The data analyzed in this study is subject to the following licenses/restrictions: the datasets used and/or analyzed during the current study are available from the corresponding author on reasonable request. Requests to access these datasets should be directed to zengkai@shzu.edu.cn.

## References

[1] Sorokin I Mamoulakis C Miyazawa K Rodgers A Talati J Lotan Y. Epidemiology of stone disease across the world. World J Urol. (2017) 35(9):1301–20. 10.1007/s00345-017-2008-6 28213860

[2] Tzelves L Türk C Skolarikos A. European association of urology urolithiasis guidelines: where are we going? Eur Urol Focus. (2021) 7(1):34–8. 10.1016/j.euf.2020.09.011 33011151

[3] Wollin DA Preminger GM. Percutaneous nephrolithotomy: complications and how to deal with them. Urolithiasis. (2018) 46(1):87–97. 10.1007/s00240-017-1022-x 29149365

[4] Knoll T Daels F Desai J Hoznek A Knudsen B Montanari E Percutaneous nephrolithotomy: technique. World J Urol. (2017) 35(9):1361–8. 10.1007/s00345-017-2001-0 28124111

[5] Okhunov Z Friedlander JI George AK Duty BD Moreira DM Srinivasan AK S.T.O.N.E. nephrolithometry: novel surgical classification system for kidney calculi. Urology. (2013) 81(6):1154–9. 10.1016/j.urology.2012.10.083 23540858

[6] Thomas K Smith NC Hegarty N Glass JM. The guy’s stone score–grading the complexity of percutaneous nephrolithotomy procedures. Urology. (2011) 78(2):277–81. 10.1016/j.urology.2010.12.026 21333334

[7] Smith A Averch TD Shahrour K Opondo D Daels FPJ Labate G A nephrolithometric nomogram to predict treatment success of percutaneous nephrolithotomy. J Urol. (2013) 190(1):149–56. 10.1016/j.juro.2013.01.047 23353048

[8] Huynh LM Huang E Patel RM Okhunov Z. Predictability and practicality of image-based scoring systems for patient assessment and outcome stratification during percutaneous nephrolithotomy: a contemporary update. Curr Urol Rep. (2017) 18(12):95. 10.1007/s11934-017-0740-5 29046986

[9] Wu WJ Okeke Z. Current clinical scoring systems of percutaneous nephrolithotomy outcomes. Nat Rev, Urol. (2017) 14(8):459–69. 10.1038/nrurol.2017.71 28534536

[10] Lai S Jiao B Jiang Z Liu J Seery S Chen X Comparing different kidney stone scoring systems for predicting percutaneous nephrolithotomy outcomes: a multicenter retrospective cohort study. Int J Surg (lond Engl). (2020) 81:55–60. 10.1016/j.ijsu.2020.07.025 32738550

[11] Peng GH Li HZ Zhang YS Zhang XB Li BC Cao MC The establishment and evaluation of SHA.LIN nephrolithometry scoring system for predicting the stone-free rate of percutaneous nephrolithotomy (in Chinese). Chin J Urol. (2015) 36(10):746–51. 10.3760/cma.j.issn.1000-6702.2015.10.006

[12] Ito Y Kikuchi E Tanaka N Miyajima A Mikami S Jinzaki M Preoperative hydronephrosis grade independently predicts worse pathological outcomes in patients undergoing nephroureterectomy for upper tract urothelial carcinoma. J Urol. (2011) 185(5):1621–6. 10.1016/j.juro.2010.12.035 21419429

[13] Asai S Fukumoto T Watanabe R Koyama K Sawada Y Noda T New classification of hydronephrosis on 18F-FDG-PET/CT predicts post-operative renal function and muscle-invasive disease in patients with upper urinary tract urothelial carcinoma. Jpn J Clin Oncol. (2018) 48(11):1022–7. 10.1093/jjco/hyy135 30252103

[14] Yarimoglu S Polat S Bozkurt IH Yonguc T Aydogdu O Aydın E Comparison of S.T.O.N.E and CROES nephrolithometry scoring systems for predicting stone-free status and complication rates after percutaneous nephrolithotomy: a single center study with 262 cases. Urolithiasis. (2017) 45(5):489–94. 10.1007/s00240-016-0935-0 27864591

[15] Memarsadeghi M Heinz-Peer G Helbich TH Schaefer-Prokop C Kramer G Scharitzer M Unenhanced multi-detector row CT in patients suspected of having urinary stone disease: effect of section width on diagnosis. Radiology. (2005) 235(2):530–6. 10.1148/radiol.2352040448 15758192

[16] Raman JD Bagrodia A Bensalah K Pearle MS Lotan Y. Residual fragments after percutaneous nephrolithotomy: cost comparison of immediate second look flexible nephroscopy versus expectant management. J Urol. (2010) 183(1):188–93. 10.1016/j.juro.2009.08.135 19913809

[17] Biswas K Gupta SK Tak GR Ganpule AP Sabnis RB Desai MR. Comparison of STONE score, guy’s stone score and clinical research office of the endourological society (CROES) score as predictive tools for percutaneous nephrolithotomy outcome: a prospective study. BJU Int. (2020) 126(4):494–501. 10.1111/bju.15130 32506712

[18] Chen K Xu K Li B Wang S Xiang S Li H. Predictive factors of stone-free rate and complications in patients undergoing minimally invasive percutaneous nephrolithotomy under local infiltration anesthesia. World J Urol. (2020) 38(10):2637–43. 10.1007/s00345-019-03070-5 31912223

[19] Karalar M Tuzel E Keles I Okur N Sarici H Ates M. Effects of parenchymal thickness and stone density values on percutaneous nephrolithotomy outcomes. Med Sci Monit. (2016) 22:4363–8. 10.12659/msm.898212 27842051 PMC5111639

[20] Jeong CW Jung JW Cha WH Lee BK Lee S Jeong SJ Seoul national university renal stone complexity score for predicting stone-free rate after percutaneous nephrolithotomy. PLoS One. (2013) 8(6):e65888. 10.1371/journal.pone.0065888 23824752 PMC3688830

[21] Labadie K Okhunov Z Akhavein A Moreira DM Moreno-Palacios J Del Junco M Evaluation and comparison of urolithiasis scoring systems used in percutaneous kidney stone surgery. J Urol. (2015) 193(1):154–9. 10.1016/j.juro.2014.07.104 25088952

[22] Choi SW Bae WJ Ha US Hong SH Lee JY Kim SW Prediction of stone-free status and complication rates after tubeless percutaneous nephrolithotomy: a comparative and retrospective study using three stone-scoring systems and preoperative parameters. World J Urol. (2017) 35(3):449–57. 10.1007/s00345-016-1891-6 27406175

[23] Noureldin YA Elkoushy MA Andonian S. Which is better? Guy’s versus S.T.O.N.E. Nephrolithometry scoring systems in predicting stone-free status post-percutaneous nephrolithotomy. World J Urol. (2015) 33(11):1821–5. 10.1007/s00345-015-1508-5 25678344

[24] Keoghane SR Cetti RJ Rogers AE Walmsley BH. Blood transfusion, embolisation and nephrectomy after percutaneous nephrolithotomy (PCNL). BJU Int. (2013) 111(4):628–32. 10.1111/j.1464-410X.2012.11394.x 22958458

[25] Akhavein A Henriksen C Syed J Bird VG. Prediction of single procedure success rate using S.T.O.N.E. Nephrolithometry surgical classification system with strict criteria for surgical outcome. Urology. (2015) 85(1):69–73. 10.1016/j.urology.2014.09.010 25530366

[26] Lehman DS Hruby GW Phillips C Venkatesh R Best S Monga M Prospective randomized comparison of a combined ultrasonic and pneumatic lithotrite with a standard ultrasonic lithotrite for percutaneous nephrolithotomy. J Endourol. (2008) 22(2):285–9. 10.1089/end.2007.0009 18208361

[27] Niall O Russell J MacGregor R Duncan H Mullins J. A comparison of noncontrast computerized tomography with excretory urography in the assessment of acute flank pain. J Urol. (1999) 161(2):534–7. 10.1016/S0022-5347(01)61942-6 9915442

[28] Wang JH Shen SH Huang SS Chang CY. Prospective comparison of unenhanced spiral computed tomography and intravenous urography in the evaluation of acute renal colic. J Chin Med Assoc: JCMA. (2008) 71(1):30–6. 10.1016/S1726-4901(08)70069-8 18218557

[29] Abou-Elela A. Epidemiology, pathophysiology, and management of uric acid urolithiasis: a narrative review. J Adv Res. (2017) 8(5):513–27. 10.1016/j.jare.2017.04.005 28748117 PMC5512151

[30] Sassanarakkit S Hadpech S Thongboonkerd V. Theranostic roles of machine learning in clinical management of kidney stone disease. Comput Struct Biotechnol J. (2023) 21:260–6. 10.1016/j.csbj.2022.12.004 36544469 PMC9755239

